# Case Report: Human Exposure to Dioxins from Clay

**DOI:** 10.1289/ehp.10594

**Published:** 2007-10-05

**Authors:** Alfred Franzblau, Elizabeth Hedgeman, Qixuan Chen, Shih-Yuan Lee, Peter Adriaens, Avery Demond, David Garabrant, Brenda Gillespie, Biling Hong, Olivier Jolliet, James Lepkowski, William Luksemburg, Martha Maier, Yvan Wenger

**Affiliations:** 1 Department of Environmental Health Sciences and; 2 Department of Biostatistics, University of Michigan School of Public Health, Ann Arbor, Michigan, USA; 3 Department of Civil and Environmental Engineering, University of Michigan College of Engineering, Ann Arbor, Michigan, USA; 4 Institute for Social Research, University of Michigan, Ann Arbor, Michigan, USA; 5 Vista Analytical Laboratory, El Dorado Hills, California, USA

**Keywords:** ball clay, clay, dioxins, furans, human exposure, polychlorinated biphenyls

## Abstract

**Context:**

For the general population, the dominant source of exposure to dioxin-like compounds is food. As part of the University of Michigan Dioxin Exposure Study (UMDES), we measured selected polychlorinated dibenzo-*p*-dioxins (PCDDs), polychlorinated dibenzofurans (PCDFs), and dioxin-like polychlorinated biphenyls (PCBs) in serum of 946 subjects who were a representative sample of the general population in five Michigan counties.

**Case presentation:**

The total toxic equivalency (TEQ; based on 2005 World Health Organization toxic equivalency factors) of serum from the index case was 211 ppt on a lipid-adjusted basis, which was the highest value observed in the UMDES study population. This subject had no apparent opportunity for exposure to dioxins, except that she had lived on property with soil contaminated with dioxins for almost 30 years, and had been a ceramics hobbyist for > 30 years. Soil from her property and clay that she used for ceramics were both contaminated with dioxins, but the congener patterns differed.

**Discussion:**

The congener patterns in this subject’s serum, soil, and ceramic clay suggest strongly that the dioxin contamination in clay and not soil was the dominant source of dioxin contamination in her serum.

Relevance to public health practice: It appears that ceramic clay, in particular the process of firing clay with unvented kilns, can be a significant nonfood and nonindustrial source of human exposure to dioxins among ceramics hobbyists. The extent of human exposure from ceramic clay is unclear, but it may be widespread. Further work is needed to more precisely characterize the routes of exposure.

Neither polychlorinated dibenzo-*p*-dioxins (PCDDs) nor polychlorinated dibenzofurans (PCDFs) were ever produced commercially in the United States, and commercial production of polychlorinated biphenyls (PCBs) in the United States stopped in 1977 [Agency for Toxic Substances and Disease Registry (ATSDR) 1994, 1998, 2000]. PCDDs and PCDFs are unintended by-products of certain chemical processes involving chlorine, as well as combustion and incineration processes. Examples include the bleaching processes involved in making white paper products, manufacture of chlorinated phenols, waste incineration, production of various metals, and combustion of fossil fuels (ATSDR 1994, 1998). Production and/or combustion of PCBs is another source of PCDFs (ATSDR 1994). Collectively referred to as dioxins or dioxin-like compounds, PCDDs, PCDFs, and PCBs became widely distributed in the environment during the 20th century largely as a result of anthropogenic activities.

For the general population, the dominant source of exposure to dioxin-like compounds is food (> 90%), primarily via consumption of dairy, meat, and fish products (ATSDR 1994, 1998, 2000). Circumstances of exposure that can be significant in selected subpopulations include occupational exposures to workers in industries that create dioxins (e.g., manufacture of phenoxyherbicides or other dioxin-contaminated chemicals and incineration operations); persons who consume large quantities of fish or game from contaminated regions; subsistence farmers who consume meat and/or dairy products produced in contaminated areas; and persons who live in the vicinity of waste incinerators. Transfer across the placenta and breast-feeding can also be important routes of exposure to fetuses and infants, respectively.

Elevated levels of PCDDs have been found in ball clay from various regions in the United States and Europe (Ferrario and Byrne 2002; Ferrario et al. 2000, 2007; Holmstrand et al. 2006). Evidence suggests that these PCDDs were formed naturally via an abiotic and nonpyrogenic process and are not the result of anthropogenic activities (Ferrario et al. 2000; Holmstrand et al. 2006). In the past, dioxin contamination from ball clay has been found in various animal products, including chicken and catfish, due to the use of ball clay as an anticaking additive in feed (Ferrario and Byrne 2000). Although contamination of food with dioxins from ball clay may have caused human exposures via the food chain, we are not aware of any reports that document ball clay as a direct source of human exposure to PCDDs, PCDFs, and/or dioxin-like PCBs.

The University of Michigan Dioxin Exposure Study (UMDES) was designed to determine whether PCDDs, PCDFs, and dioxin-like PCBs (hereafter collectively referred to as “dioxins”) in soil and/or house dust are related to or explain serum levels of these contaminants, with adjustment for other known risk factors (i.e., diet, occupation, age, body mass index, etc.). The study was undertaken in response to concerns among the population of Midland and Saginaw Counties that dioxin-like compounds from the Dow Chemical Company facilities in Midland, Michigan, have contaminated areas of the City of Midland and sediments in the Tittabawassee River flood plain. The study measured the serum levels of the World Health Organization (WHO) 29 dioxin congeners with consensus toxic equivalency factors (TEFs) in a random sample of the population in the study regions (Van den Berg et al. 2006). Analyzable serum samples were obtained from 946 participants. Eligible subjects also had the same congener analyses performed on soil samples from around their homes (*n* = 766) and on house dust sampled from inside homes (*n* = 764). All chemical analyses for PCDDs, PCDFs, and PCBs were performed by Vista Analytical Laboratory (El Dorado Hills, CA) using modified U.S. Environmental Protection Agency (EPA) methods 8290 (U.S. EPA 1994) and 1668, Revision A (U.S. EPA 1999).

As part of a follow-up investigation of high serum dioxin outliers, eight subjects with the highest toxic equivalency (TEQ) in serum (i.e., > 2.5 studentized residuals above the mean of the log-transformed serum TEQ results after adjustment for age, age^2^, and body mass index) completed open-ended semistructured interviews in an effort to better understand why these subjects had such high levels of dioxins in their serum (Franzblau et al. 2006). Briefly, it was found that most of the subjects reported frequent and prolonged consumption of wild game and/or sport-caught fish; high outlier serum levels did not appear to be related to contamination of soil or house dust, occupation, activities in the contaminated areas of the region, or proximity to incinerators. In addition, two subjects reported substantial weight loss, which may have also contributed to the unusually elevated levels of dioxins in their serum. However, the subject who had the highest serum TEQ in the entire study, 211 ppt, did not fit these patterns. The median serum TEQ for the entire study (*n* = 946 subjects) was 19.6 ppt; the 95th percentile was 58.6 ppt. Here, we report the results of further investigations into why this subject had elevated levels of dioxins in her serum.

## Case Presentation

The index case (case 1) is female and was 77 years of age at the time her blood was sampled. She had lived along the Tittabawassee River for almost 30 years, downstream from the Dow plant located in Midland, Michigan. Her total serum TEQ (211 ppt) was the highest among 946 randomly selected subjects in the UMDES study who had serum tested. All study participants provided written informed consent that had been approved by the University of Michigan Institutional Review Board.

Case 1 denied any occupational history that might suggest potential opportunity for exposure to dioxins for herself or anyone else who had lived in her household. She denied consumption of wild game since she was a child. Her consumption of sport-caught fish, which had ended approximately 13 years earlier, consisted of approximately one meal per day during a 2-week vacation in rural Canada each year for 20 years. She denied ever eating fish from the Tittabawassee River or the Saginaw River. She never prepared or ate store-bought fish at home, but in the 1960s and 1970s she would eat about one fish meal per month at local restaurants (she believes that the restaurant fish was from outside the region). She never resided in the vicinity of industrial incinerators. She is a lifelong nonsmoker, and she denied any recent change in body weight. She did not garden on the property, and she never ate vegetables grown on the property.

Soil collected from the perimeter of the house (about 80 m from the river) had a total TEQ of 18 ppt. The median background level of dioxins in soil in the lower peninsula of Michigan is 4.6 ppt, and the 97.5th percentile is 34 ppt. Soil obtained from her property immediately adjacent to the Tittabawassee River (i.e., a flood plain sample) had a total TEQ of 397 ppt, and the congener pattern was dominated by PCDFs in a pattern that was typical of the contamination found in the Tittabawassee river flood plain downstream from Midland (Hilscherova et al. 2003). The total TEQ of the house dust was 85 ppt. Background levels for dioxins in house dust in the control area of the UMDES were as follows: median, 14 ppt; 75th percentile, 35 ppt, and 95th percentile, 263 ppt. The congener pattern in house dust was dominated by the higher chlorinated dioxins, with low concentrations of PCDFs (Table 1).

Along with a group of friends, she had been very involved in ceramics as a hobby from the early 1960s up to about the mid-1990s. She purchased ceramic clay in liquid form (“slip”), and poured this into molds to harden. She never added anything to the liquid clay, except for distilled water on occasion. When the wet clay had hardened sufficiently, she removed the piece (“green pottery”) from the mold and let it dry further. The molds were made of plaster, and she denied ever using organic solvents to clean molds. Rough edges of the green pottery were smoothed with a wet sponge or sometimes sanded. She performed ceramics work on average about three afternoons or evenings per week for about three decades. She never used gloves or any respiratory protection. She fired the pottery in one of three unvented electric kilns in the basement of her house. The peak kiln temperature normally attained was approximately 1,800°F (cone number 6). After the first firing, she painted the pieces with various glazes and then re-fired them at the same temperature. She stopped doing ceramics 11–12 years before blood sampling.

Results of chemical analyses of her serum, house dust, and representative samples of soil collected from her property are shown in Table 1. The serum, house dust, and soil samples were analyzed as part of the main UMDES study. Approximately 1 year later, as part of the outlier follow-up investigation, one randomly selected sample each of the subject’s fired clay (unglazed), unfired clay (unglazed), and liquid clay were sent for chemical analyses to the same laboratory that performed all analyses for the UMDES (Vista Analytical Laboratory). Results of analyses of the three ceramic clay samples are also shown in Table 1, along with published data on dioxins in ball clay (Ferrario and Byrne 2002).

As noted above, the index case did ceramics with an informal group of friends. Two of these friends were still alive, and both agreed to be interviewed and to provide blood samples for analyses of dioxins (cases 2 and 3). No soil or dust samples were collected in relation to these two cases.

At the time of interview, case 2 was 85 years of age, and case 3 was 83 years of age. Like the index case, they had no opportunity for occupational exposure to dioxins. They did not live adjacent to the Tittabawassee River or near any industrial incinerators. They denied fishing or regular consumption of fish from the Tittabawassee River, the Saginaw River, or Saginaw Bay, and they also denied regular consumption of sport-caught fish from elsewhere. They denied consumption of wild game. They were also nonsmokers, and they denied any recent change in body weight.

The time frame, frequency, and duration and manner of ceramics work were approximately the same for cases 2 and 3 as for the index case. A distinction was that case 1 had three kilns in her basement, whereas the other two cases had only one kiln each, they used the kilns less frequently, and the kilns were located in garages, not in the basement or elsewhere inside their homes.

**Figure 1:**
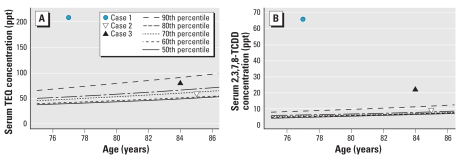
Serum TEQ (*A*) and serum 2,3,7,8-TCDD (*B*) for cases with quantile curves based on female controls from Jackson and Calhoun Counties.

Results of chemical analyses of serum for cases 2 and 3 are also shown in Table 1, and they are plotted in Figure 1 (along with results from case 1). Although the total serum TEQs and the serum mass concentrations for TCDD for cases 2 and 3 are elevated compared with those of the controls, they are substantially lower than for case 1.

## Discussion

**Figure 2:**
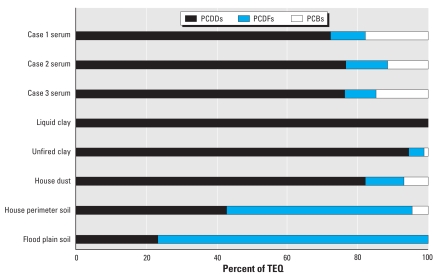
Relative contribution of PCDDs, PCDFs, and dioxin-like PCBs to TEQ for serum, clay, house dust, and soil results.

The overall pattern of results shown in Table 1 and Figure 2, in particular the high PCDD:PCDF ratio in case 1’s serum and clay, suggest strongly that the dioxin contamination in the ceramic clay, and not the dioxin contamination in soil from her property, was the dominant source of dioxin contamination in this subject’s serum. The overall congener profile of PCDDs, PCDFs, and PCBs in the serum of case 1 is different from the pattern seen in other subjects from the UMDES who had high total serum TEQ. Among the other UMDES subjects with the highest serum TEQs, PCBs were the dominant contaminants (i.e., > 50% of the TEQ in most cases was attributable to PCBs), along with lower chlorinated dioxins; unlike case 1, these other subjects reported diets rich in wild game and/or sport-caught fish (Franzblau et al. 2006).

There are a number of possible pathways by which the dioxins in the ceramic clay may have gotten into the body of case 1: *a*) direct absorption of dioxins through her skin while handling liquid clay or unfired ceramics; *b*) inhalation of dioxins volatilized when ceramic pieces were fired in the unvented kilns in her basement; *c*) ingestion of clay or clay particles that landed on food items in her house, or during food handing or by contact between the hands and the mouth; *d*) inhalation of clay dust from handling and sanding unfired ceramic items; and *e*) inhalation of clay dust that became mixed with house dust. On the basis of multivariate models from the UMDES study, we do not believe that the last pathway is significant: Dioxins in house dust are not a major source of dioxins in serum of household residents. Similar models also demonstrate that soil contamination around the home is not a major source of dioxins in serum (Garabrant et al. 2006). Fired ceramics contain very little dioxin and do not appear to be a source of exposure. Cases 2 and 3 handled ceramic clay in a manner that was similar to that of case 1, but their TEQ and 2,3,7,8-TCDD levels in serum were dramatically lower than hers. The major distinction appears to be that cases 2 and 3 each had only one kiln, which were used less frequently, and the kilns were located in garages, not in the basement or elsewhere inside the living space of their homes. Although the number of subjects is small, these results suggest that the dominant route of exposure for case 1 was inhalation of dioxins volatilized during firing of ceramic pieces in the unvented kilns in the basement of her home. The fact that cases 2 and 3 had above-average TEQ and 2,3,7,8-TCDD levels in their serum (after adjustment for age) could be due to their more limited exposure to kilns and/or a limited role for exposure from direct handing of clay materials.

Ball clay is sedimentary in origin, and it is usually composed of kaolinite, mica, and quartz. However, “ball clay” is in part a term of art or industry rather than a purely mineralogical term. The name derives from the original practice of mining such clay in cubes that would become rounded into balls during handling and storage, and hence was referred to as “ball clay” (Industrial Minerals Association-North America 2007).

In 2004, just over 1.2 million metric tons of ball clay were mined in the United States (Virta 2004). Tennessee accounted for 62% of production, with the remainder coming from Texas, Mississippi, and Kentucky, in decreasing order of production; a negligible amount is also mined in Indiana (Virta 2004). Major uses include floor and wall tile (35%), sanitary ware (26%), and miscellaneous ceramics (17%; includes catalysts, electrical porcelain, fiberglass, fine china/dinnerware, glass, mineral wool, roofing granules, and miscellaneous ceramics) (Virta 2004). Pottery accounts for only 2% of all tonnage. Ceramics are made from all types of clay, but ball clay accounts for 44% of clay used in production of ceramics products. As noted above, some ball clays from the United States have been shown to be contaminated with dioxins (Ferrario and Byrne 2002; Ferrario et al. 2007). Our subjects reported that they purchased clay from regional retail sales outlets, but the precise geological source of the clay used by our subjects is not known. It is uncertain whether their clay was composed of ball clay known to be contaminated, or whether it came from other sources not previously shown to be contaminated with dioxins.

Ferrario and Byrne (2002) reported on levels of PCDDs in ball clay. They also measured PCDFs, but they stated that no PCDFs were detected above the limit of detection (LOD; all < 2.0 ppt). Ferrario and Byrne (2002) did not mention measurements of PCBs. In the present study, our analyses indicate that the unfired clay used by our subjects had measurable levels of all 12 PCBs that have TEFs, particularly PCB-77, PCB-105, PCB-118, and PCB-156. The unfired clay sample also had measurable, although not extreme levels, of PCDFs. In contrast, the congener pattern in the liquid clay sample was similar to the published pattern for ball clay, with essentially no measurable PCDFs or PCBs. As noted above, our cases denied ever adding anything except distilled water to the liquid clay, so the origin of the PCDFs and PCBs in the unfired clay sample is unclear.

Previous studies have identified a 1,2,3,6,7,8/1,2,3,7,8,9 hexachlorinated dibenzo-*p*-dioxin (HexaCDD) congener ratio < 1 as a distinctive characteristic of ball clay (Ferrario et al. 2000, 2007). The ratio in our liquid clay and unfired clay samples is similar to what has been reported previously (Table 1). However, the corresponding ratios for serum from all three cases, and also dust and soil samples in the present study, all have a ratio > 1. The results for serum from all 946 subjects in the UMDES are similar to the three cases in the present study (i.e., the mean 1,2,3,6,7,8/1,2,3,7,8,9 HexaCDD congener ratio for all subjects in the UMDES study was 6.28; median, 6.10; range, 1.87–13.29). Previous studies in other species (i.e., chickens and fish) have documented that a HexaCDD ratio < 1 found in ball clay was conserved in the tissues from these species that had been fed ball clay (Ferrario and Byrne 2000; Ferrario et al. 2000). The explanation for the ratio being > 1 in the serum of our three subjects is unclear. It could be that mammalian uptake and/or metabolism differs from that in nonmammalian species. The dioxin exposure of our three cases may have been influenced by the fact that they were exposed by means of volatilization of the dioxins at high temperature, and the congener pattern may have been altered by the high temperature. An American market basket study of beef, pork, and chicken indicated that the 1,2,3,6,7,8/1,2,3,7,8,9 HexaCDD ratio is > 1 in the food supply, and, as previously noted, food is the dominate source of exposure for most people (ATSDR 1994, 1998, 2000; Huwe and Larsen 2005). These results and observations suggest that that a ball-clay feeding/exposure study conducted with mammals, or a study of serum from workers known to be exposed to ball clay, could be useful in furthering our understanding of human exposure to the dioxins in ball clay.

## Relevance to Public Health Practice

We are not aware of any previous demonstration of human exposure to dioxins related to making ceramics. The magnitude of the public health significance of our findings is not clear, but the number of people exposed to dioxins in clay could vary considerably. We do not know what fraction of clays used in school art classes, by ceramics enthusiasts, by professional potters, or in commercial operations is contaminated with dioxins, and the extent of the contamination may vary. We also do not know how many individuals, art studios, and commercial operations have kilns, the operational characteristics of these kilns, and how the kilns are vented, if at all. Further investigations are warranted to better determine routes of exposure, in particular to confirm whether volatilization of dioxins during firing is the most important route of exposure, and also to determine the extent of dioxin contamination of clay used by ceramicists and in commercial operations.

Our results suggest that clay, in particular firing clay with unvented kilns, can be a significant nonindustrial source of human exposure to dioxins among ceramics hobbyists. Further work is needed to more precisely characterize the route(s) of exposure.

## Figures and Tables

**Table 1:** Concentrations (ppt) of PCDDs, PCDFs, and PCBs in serum, house dust, soil, and clay, and published concentrations for ball clay.

		Serum concentration			Case 1			Liquid clay			Processed clay			
Compound	WHO 2005TEF^a^	Case 1	Case 2	Case 3	House dust	House perimeter	Flood plain	Wet	Unfired	Fired	Wet ball clay^b^	Mixture^b^	Unfired^b^	Fired^b^
PCDDs														
2,3,7,8-TCDD	1	65.4	9	22.1	2.49	2.67	65	31	5.34	0.05^c^	1,480	191	212	0.1
1,2,3,7,8-PentaCDD	1	59.8	17	18.4	2.85	2.52	10.6	85	46.1	0.15	1,220	155	157	0.4
1,2,3,4,7,8-HexaCDD	0.1	30.8	12.1	17.5	5.98	2.42	8.7	86.5	44.7	0.14^c^	271	32	30	0.4
1,2,3,6,7,8-HexaCDD	0.1	189	83.6	82.3	84.7	6.36	58.6	142	63.5	0.28	777	103	93	0.4
1,2,3,7,8,9-HexaCDD	0.1	32.4	10.7	14.1	31	4.66	12.9	454	388	0.28	2,890	395	363	0.4
1,2,3,4,6,7,8-HeptaCDD	0.01	149	74.7	57.1	4,620	110	652	2,430	1,280	1.92	7,500	1,130	1,080	0.4
OctaCDD	0.0003	541	914	615	20,900	851	5,800	48,500	18,400	7.26	97,900	29,700	23,000	1.4
PCDFs														
2,3,7,8-TetraCDF	0.1	1.09	0.264^c^	0.716	9.96	20	836	0.07^c^	11	0.09^c^	ND	ND	ND	ND
1,2,3,7,8-PentaCDF	0.03	0.4^c^	0.141^c^	0.533	6.85	12	543	0.08^c^	17.5	0.21	ND	ND	ND	ND
2,3,4,7,8-PentaCDF	0.3	50	12.4	13.7	7.97	13.7	442	0.07^c^	7.88	0.13^c^	ND	ND	ND	ND
1,2,3,4,7,8-HexaCDF	0.1	27	8.46	10	10.4	12.2	375	0.07^c^	4.73	0.08^c^	ND	ND	ND	ND
1,2,3,6,7,8-HexaCDF	0.1	24.7	8.56	7.96	7.73	5.36	126	0.50	5.2	0.16	ND	ND	ND	ND
1,2,3,7,8,9-HexaCDF	0.1	1.06^c^	0.397^c^	0.356^c^	2.11	3.06	80.4	0.15^c^	1.67	0.07^c^	ND	ND	ND	ND
2,3,4,6,7,8-HexaCDF	0.1	4.23	1.63	1.33	6.79	5.85	48.7	0.1^c^	1.7	0.13	ND	ND	ND	ND
1,2,3,4,6,7,8-HeptaCDF	0.01	9.45	5.24	6.73	289	53.5	771	0.16	3.29	0.62	ND	ND	ND	ND
1,2,3,4,7,8,9-HeptaCDF	0.01	0.68^c^	0.257^c^	0.505^c^	9.4	3.41	65	0.07^c^	1.94	0.08	ND	ND	ND	ND
OctaCDF	0.0003	2.1^c^	1.04^c^	1.06	636	92.6	1,740	4.57	5.27	0.34	ND	ND	ND	ND
PCBs														
PCB-81	0.0003	9.33	1.18^c^	3.77	40.2	1.58	16.1	0.15^c^	221	9.27	NR	NR	NR	NR
PCB-77	0.0001	6.39	2.36	5.72	869	17.9	258	0.42	800	18.3	NR	NR	NR	NR
PCB-126	0.1	309	30	66.5	48.9	7.77	9.5	0.25^c^	9.41	0.58	NR	NR	NR	NR
PCB-169	0.03	116	43.5	51.8	2.03	1.16	2.2	0.19^c^	0.6^c^	0.07^c^	NR	NR	NR	NR
PCB-105	0.00003	9,220	3,360	7,320	6,970	170	492	1.45	3,130	213	NR	NR	NR	NR
PCB-114	0.00003	4,620	2,220	2,400	455	7.36	34.7	0.25^c^	214	8.5	NR	NR	NR	NR
PCB-118	0.00003	60,100	19,200	33,100	16,500	286	1,080	4.19	8,000	345	NR	NR	NR	NR
PCB-123	0.00003	1,560	270	523	417	10.1	27	0.24^c^	118	5.38	NR	NR	NR	NR
PCB-156	0.00003	21,500	14,000	13,700	1,570	68.6	110	0.21	1,390	25.5	NR	NR	NR	NR
PCB-157	0.00003	5,200	2,990	2,890	332	18.9	26.8	0.08^c^	98.2	5.07	NR	NR	NR	NR
PCB-167	0.00003	7,350	3,300	3,440	682	33.4	47	0.09^c^	575	7.15	NR	NR	NR	NR
PCB-189	0.00003	1,920	800	775	103	9.19	15	0.06^c^	215	1.44	NR	NR	NR	NR
Total TEQ		211	49	69	85	18	397	223	126	0.5	3,190	419	435	< 1

Abbreviations: ND, not detected (< LOD); NR, not reported. Serum results are reported on a lipid-adjusted basis; all other results are reported on a dry-weight basis.

a Data from Van den Berg et al. (2006).

b Data from Ferrario and Byrne (2002).

c < LOD; concentrations were substituted with 
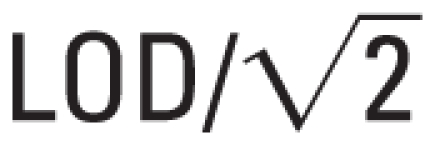
.
